# Regulatory effect of two *Trichinella spiralis* serine protease inhibitors on the host’s immune system

**DOI:** 10.1038/s41598-019-52624-5

**Published:** 2019-11-19

**Authors:** Jingyun Xu, Pengcheng Yu, Lijia Wu, Mingxu Liu, Yixin Lu

**Affiliations:** Heilongjiang Key Laboratory for Zoonosis, College of Veterinary Medicine, Northeast Agricultural University, 600 Changjiang Street, Harbin, 150030 China

**Keywords:** Autoimmunity, Inflammation

## Abstract

*Trichinella spiralis* (*T. spiralis*) is widely distributed throughout the world and can cause serious zoonotic parasitic diseases. Serine protease inhibitors (SPIs) have unique enzyme inhibitory activity and occupy an important position in the interaction between parasites and hosts. In order to further understand the immunoprotective effect of SPIs on *T. spiralis* invasion *in vivo*, the Kazal and Serpin type SPI of *T. spiralis* (TsKaSPI and TsAdSPI) were mixed with Freund’s adjuvant in equal volume to immunize mice. The results showed that the expression of IgG1 and IgG2a in serum, the proliferation of spleen cells, and the expression level of cytokines were all increased. The results of flow cytometry showed that the expression of CD4+CD25+Foxp3+ Tregs, CD8+CD28− T cells, CD19+CD5+CD1d^hi^ Bregs in spleen were also increased. Therefore, both TsKaSPI and TsAdSPI could induce strong humoral and cellular immune responses. And the results of adult reduction rate and pathological changes of intestine after adult invasion also indicated that both TsKaSPI and TsAdSPI could prevent *T. spiralis* from invading intestine. To explore the regulatory effects of TsKaSPI and TsAdSPI on the immune function of macrophage, the results of ELISA showed that the expression of cytokines in cell supernatant were increased. And the results of Western blot showed that both TsKaSPI and TsAdSPI could induce phosphorylation of JAK2 and STAT3 receptors, thereby affecting the signal transduction of macrophages. This experiment demonstrated that SPIs could act as effector molecules affecting the immune function of host when infected with *T. spiralis*.

## Introduction

*Trichinella spiralis* (*T. spiralis*) is a nematode that establishes persistent infection in the muscle of mammals and can cause very serious zoonotic parasitic diseases^1,2^. It is one of the most widely distributed zoonotic pathogens in the world. *T. spiralis* has a wide range of hosts in nature, and ability to complete parasitism in a variety of animals. Moreover, the different developmental phase of *T.spialis* all occurs in a single host, causing severe damage of the host, so its mechanism of evading the host’s immune system has attracted extensive attention. When *T. spiralis* establishes a parasitic relationship with the host, it will generate various immune evasion mechanisms, so that it can successfully parasitize and minimize the damage of the host. In the early phase of *T. spiralis* infection, it can induce Th1/Th2 mixed immune response in the host, and mainly based on Th2 type^3^. The main manifestations are increased IgG and cytokines level, as well as increased eosinophils and basophils, which can help the host to resist infection. For a long time, scholars have been studying the key components of *T. spiralis* that play an important role in immune evasion, and *T. spiralis* serine protease inhibitors (SPIs) can inhibit a variety of intestinal digestive enzymes of the host, it has been identified as the major regulatory antigen in the process of *T. spiralis* invading the host^4,5^. Therefore, the study on its structure and function is of great significance.

SPI is an enzyme activity regulator with conserved amino acid sequence and special spatial structure. It can inhibit target enzymes by changing its own conformation, and involved in many basic life activities, such as cell migration, tumor inhibition, inflammatory reaction, protein folding, cell matrix reconstruction^6,7^. Studies have shown that parasite SPI has unique enzyme inhibitory activity, which can protect the parasite against the digestion of the host’s digestive enzymes, and provide favorable conditions for the parasite to survive, develop, migrate and settle in the host, help the parasite to resist the host’s immune response^8–11^.

Our laboratory has obtained active recombinant *T. spiralis* SPIs (TsKaSPI, TsAdSPI) by prokaryotic expression. Ma *et al*.^12^ found that TsKaSPI can inhibit the activity of trypsin, elastase, chymotrypsin, cathepsin G, thrombin and granulase B. TsAdSPI also inhibited the activity of trypsin, elastase and chymotrypsin^13^. Some researchers believe that in the intestinal phase of *T. spiralis* infection, SPI will not induce the host to produce specific antibodies, but will rapidly bind to multiple proteases in the intestine, the autoimmune sites can be quickly masked, thereby reducing the responsiveness of the intestinal phase and playing a role in immune evasion^14,15^. In parenteral phase, the antigen sites of SPI exposed, and play a role in immune evasion by regulating multiple molecules of the immune system. The regulation of the immune system by *T. spiralis* SPI is gradually being revealed, and the purpose of this study is to investigate its regulatory effect on host’s immune system during *T. spiralis* invasion.

## Result

### Spleen cells proliferation

**Figure 1:**
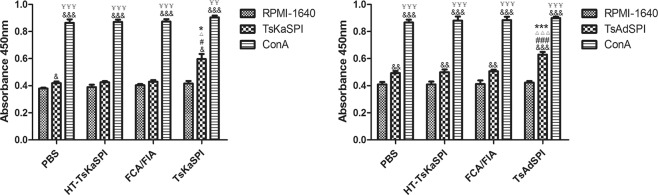
Proliferation of spleen cells extracted from each group after ConA, RPMI-1640, recombinant protein stimulation *in vitro*. Data are shown as mean ± SD of 3 mice per group. ^#^*P* < 0.05, ^##^*P* < 0.01, ^###^*P* < 0.001 versus PBS group; ^△^*P* < 0.05, ^△△^*P* < 0.01, ^△△△^*P* < 0.001 versus HT-TsKaSPI group; ^*^P < 0.05, ^**^P < 0.01, ^***^P < 0.001 versus FCA/FIA group; ^§^*P* < 0.05, ^§§^*P* < 0.01, ^§§§^*P* < 0.001 for TsKaSPI vs TsAdSPI; ^&^*P* < 0.05, ^&&^*P* < 0.01, ^&&&^*P* < 0.001 for ConA, TsKaSPI/TsAdSPI vs R*P*MI-1640 in the same group; ^¥^*P* < 0.05, ^¥¥^*P* < 0.01, ^¥¥¥^*P* < 0.001 for ConA vs TsKaSPI/TsAdSPI in the same group.

ConA has a potent effect on promoting mitosis and lymphocyte transformation, so stimulated with ConA *in vitro* was selected as positive control. The results showed that the number of spleen cells extracted from the PBS, HT-TsKaSPI, FCA/FIA, TsKaSPI and TsAdSPI group were significantly increased after ConA stimulation, compared with RPMI-1640, TsKaSPI and TsAdAPI stimulation (*P* < 0.001). The spleen cells extracted from the TsKaSPI (*P* < 0.05) and TsAdSPI group (*P* < 0.01) were stimulated with the corresponding proteins *in vitro*, and the number of spleen cells in the two groups were significantly higher than that in the PBS, HT-TsKaSPI and FCA/FIA group (Fig. 1).

### Changes of expression of CD4+CD25+Foxp3+ Treg cells, CD8+CD28− T cells and CD19+CD5+CD1dhi Breg cells in spleen

**Figure 2:**
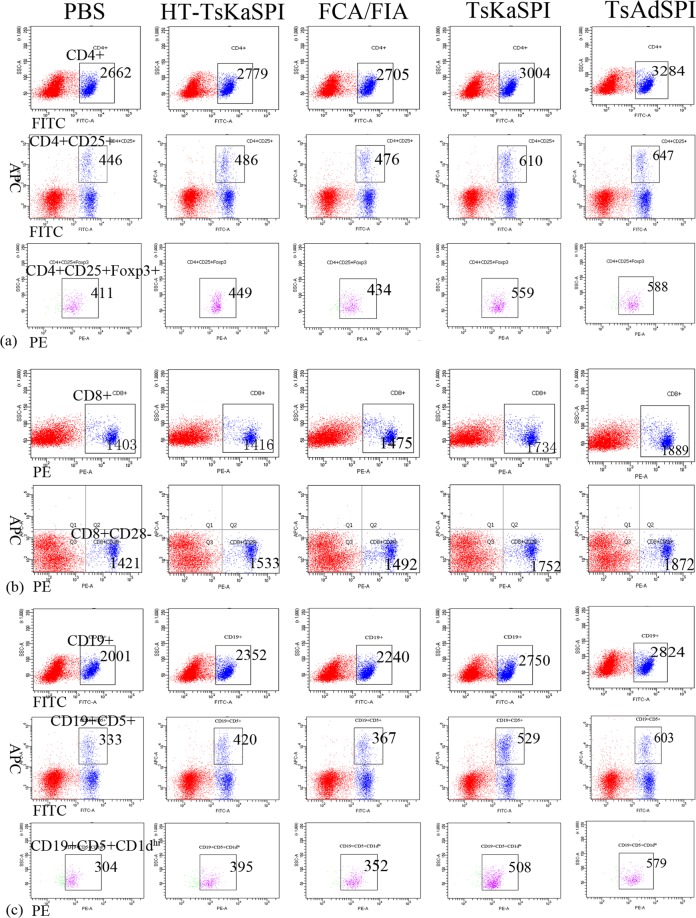
Demonstration of the gating strategy for the flow cytometric analysis of mouse CD4 CD25+Foxp3+ Treg (a), CD8+CD28− T cell (b), CD19+CD5+CD1d^hi^ Breg (c) from spleen. In this experiment a single cell suspension was prepared from the spleen of each group and stained with CD4 (FITC), CD25 (APC), Foxp3 (PE), CD8 (PE), CD28 (APC), CD19 (FITC), CD5 (APC), CD1d (PE) based on surface and intracellular staining protocols, respectively. Data were collected with FACSDiva flow cytometer and analyzed. Lymphocytes are identified by their scatter properties (FSC-A × SSC-A plot).

Mice were sacrificed 7 days after the third immunization, and prepared spleen lymphocyte single cell suspension. Flow cytometry was performed to detect CD4+CD25+Foxp3+ Treg cells (Fig. 2(a)), CD8+CD28− T cells (Fig. 2(b)) and CD19+CD5+CD1d^hi^ Breg cells (Fig. 2(c)) in the spleen of each group. And the ratio of CD4+/CD8+ T cells in each group was calculated.

**Figure 3:**
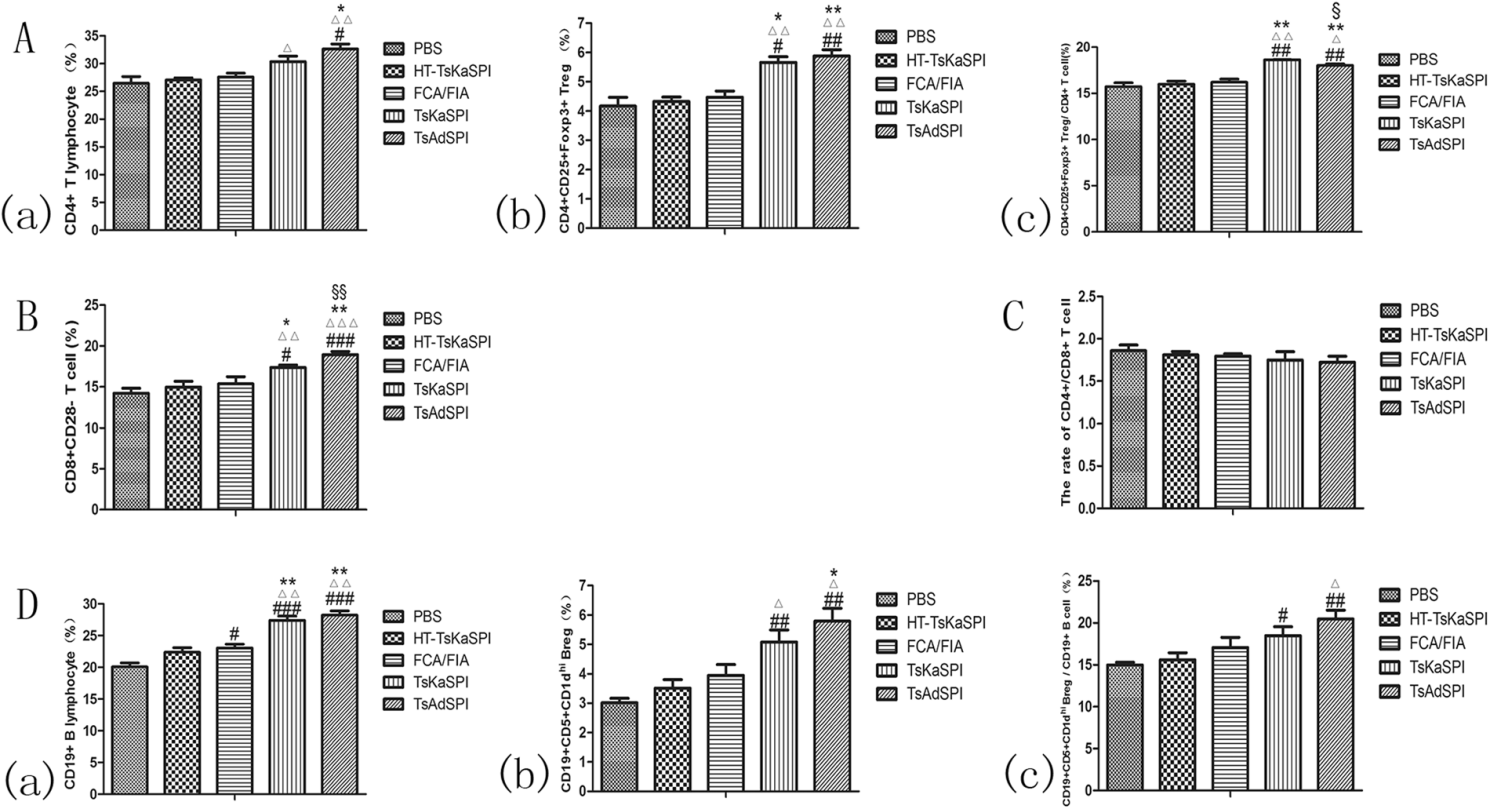
The percentage of CD4+ T lymphocytes in the total lymphocytes in the gate (a), the percentage of CD4+CD25+Foxp3+ Tregs in the total lymphocytes in the gate (b) and the percentage of CD4+ T lymphocytes in CD4+CD25+Foxp3+ Tregs (c) in spleens of five groups were showed in (**A**); the percentage of CD8+CD28− T cells in the total lymphocytes in the gate were showed in (**B**); the rate of CD4+/CD8+ T cells in spleens of five groups were showed in (**C**); and the percentage of CD19+ B lymphocytes in the total lymphocytes in the gate (a), the percentage of CD19+CD5+CD1d^hi^+ Bregs in the total lymphocytes in the gate (b) and the percentage of CD19+ B lymphocytes in CD19+CD5+CD1d^hi^ Bregs (c) in spleens of five groups were showed in (**D**). Data are shown as mean ± SD of 3 mice per group. ^#^*P* < 0.05, ^##^*P* < 0.01, ^###^*P* < 0.001 versus PBS group; ^△^*P* < 0.05, ^△△^*P* < 0.01, ^△△△^*P* < 0.001 versus HT-TsKaSPI group; ^*^P < 0.05, ^**^P < 0.01, ^***^P < 0.001 versus FCA/FIA group; ^§^*P* < 0.05, ^§§^*P* < 0.01, ^§§§^*P* < 0.001 for TsKaSPI vs TsAdSPI.

The results showed that the percentage of CD4+ T lymphocytes in the total number of cells in the gate in the TsAdSPI group (32.62 ± 1.58) was significantly higher than that in the PBS group (26.45 ± 2.09) (*P* < 0.05), HT-TsKaSPI group (27.05 ± 0.68) (*P* < 0.01) and FCA/FIA group (27.58 ± 1.22) (*P* < 0.05). While there was no significant difference compared the TsKaSPI group with PBS group and FCA/FIA group (Fig. 3A(a)); The percentage of CD4+CD25+Foxp3+ Treg cells in the total number of cells in the gate was significantly higher in the TsKaSPI group (5.66 ± 0.33) and TsAdSPI group (5.88 ± 0.37) than that in the PBS group (4.17 ± 0.51), HT-TsKaSPI group (4.33 ± 0.26) and FCA/FIA group (4.47 ± 0.36) (Fig. 3A(b)); At the same time, the percentage of CD4+CD25+Foxp3+ Treg in CD4+ T lymphocytes was calculated. The results showed that it was significantly higher in the TsKaSPI group (18.64 ± 0.04) and TsAdSPI group (18.03 ± 0.27) than that in the PBS group (15.73 ± 0.73), HT-TsKaSPI group (15.99 ± 0.57) and FCA/FIA group (16.21 ± 0.58), and the percentage of TsKaSPI group was significantly higher than that of TsAdSPI group (*P* < 0.05) (Fig. 3A(c)); For CD8+CD28− T cells, the percentage of it in total lymphocytes in the gate in the TsAdSPI group (18.95 ± 0.38) was significantly higher than the PBS group (14.21 ± 0.62) (*P* < 0.001), HT-TsKaSPI group (14.97 ± 0.72) (*P* < 0.001), FCA/FIA group (15.37 ± 0.87) (*P* < 0.01) and TsKaSPI group (17.37 ± 0.30) (*P* < 0.01), and the TsKaSPI group was significantly higher than the PBS group (*P* < 0.05), HT-TsKaSPI group (*P* < 0.01) and FCA/FIA group (*P* < 0.05) (Fig. 3B); The ratio of CD4+/CD8+ T cells was found to be lower in the TsKaSPI group (1.75 ± 0.10) and TsAdSPI group (1.72 ± 0.07) than that in the PBS group (1.86 ± 0.07), but there was no significant difference between the groups (Fig. 3C);

The percentage of CD19+ B lymphocytes in total lymphocytes was significantly higher in the TsKaSPI group (27.39 ± 1.15) and TsAdSPI group (28.21 ± 1.20) than that in the PBS group (20.17 ± 1.08) (*P* < 0.001), HT-TsKaSPI group (22.37 ± 1.25) (*P* < 0.01) and FCA/FIA group (23.03 ± 1.05) (*P* < 0.01), and there was no significant difference between the two groups (Fig. 3D(a)); The percentage of CD19+CD5+CD1d^hi^ Breg in the TsKaSPI group (5.08 ± 0.71) and TsAdSPI group (5.79 ± 0.77) was significantly higher than that in the PBS group (3.01 ± 0.26) (*P* < 0.01) and HT-TsKaSPI group (3.51 ± 0.51) (*P* < 0.05), and the percentage in the TsAdSPI group was significantly higher than that in the FCA/FIA group (3.95 ± 0.64) (*P* < 0.05) (Fig. 3D(b)); Therefore, the percentage of CD19+CD5+CD1d^hi^ Breg in CD19+ B lymphocytes was calculated, and the results showed that it was significantly higher in the TsKaSPI group (18.50 ± 1.82) (*P* < 0.05) and TsAdSPI group (20.46 ± 1.84) (*P* < 0.01) than PBS group (14.99 ± 0.53), and that in the TsAdSPI group was significantly higher than the HT-TsKaSPI group (15.62 ± 1.41) (*P* < 0.05), but there was no significant difference among other groups (Fig. 3D(c)).

### Effects of two recombinant proteins on IgG subtypes

**Figure 4:**
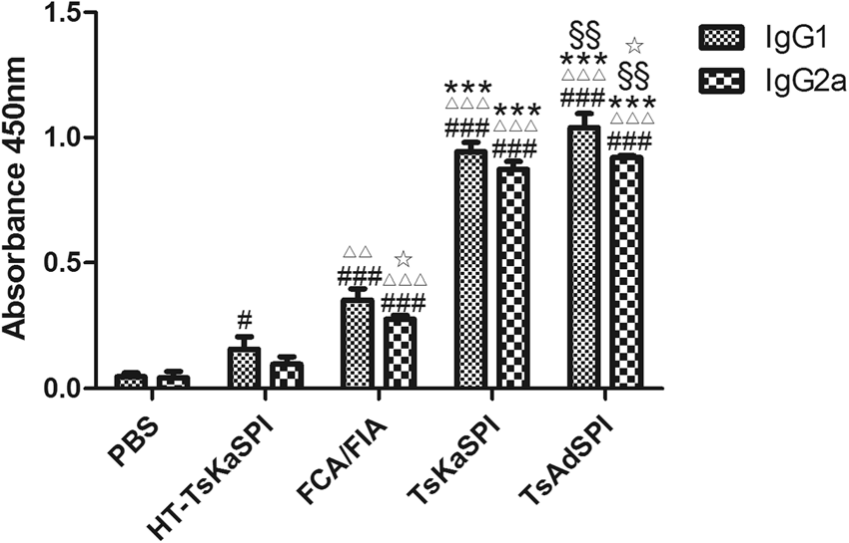
Analyze changes of IgG subtypes: IgG1 and IgG2a. Data are shown as mean ± SD of 3 mice per group. ^#^*P* < 0.05, ^##^*P* < 0.01, ^###^*P* < 0.001 versus PBS group; ^△^*P* < 0.05, ^△△^*P* < 0.01, ^△△△^*P* < 0.001 versus HT-TsKaSPI group; ^*^P < 0.05, ^**^P < 0.01, ^***^P < 0.001 versus FCA/FIA group; ^§^*P* < 0.05, ^§§^*P* < 0.01, ^§§§^*P* < 0.001 for TsKaSPI vs TsAdSPI; ^☆^*P* < 0.05, ^☆☆^*P* < 0.01, ^☆☆☆^*P* < 0.001 for IgG1 vs IgG2a in the same group.

Mice were sacrificed 7 days after the third immunization, and serum samples of each group were collected to analyze the changes of IgG subtypes: IgG1 and IgG2a. The results were shown in Fig. 4. The expression level of IgG1 in the TsKaSPI group (0.94 ± 0.04) and TsAdSPI group (0.87 ± 0.03) were significantly higher than that in the PBS group (0.05 ± 0.02), HT-TsKaSPI group (0.16 ± 0.05) and FCA/FIA group (0.35 ± 0.05) (*P* < 0.001), and the TsAdSPI group was significantly higher than the TsKaSPI group (*P* < 0.01); Meanwhile the expression level of IgG2a in each group was similar to IgG1; however, there was significantly difference between IgG1 and IgG2a in FCA/FIA group and TsAdSPI group (*P* < 0.05).

### Changes in the expression of cytokine

**Figure 5:**
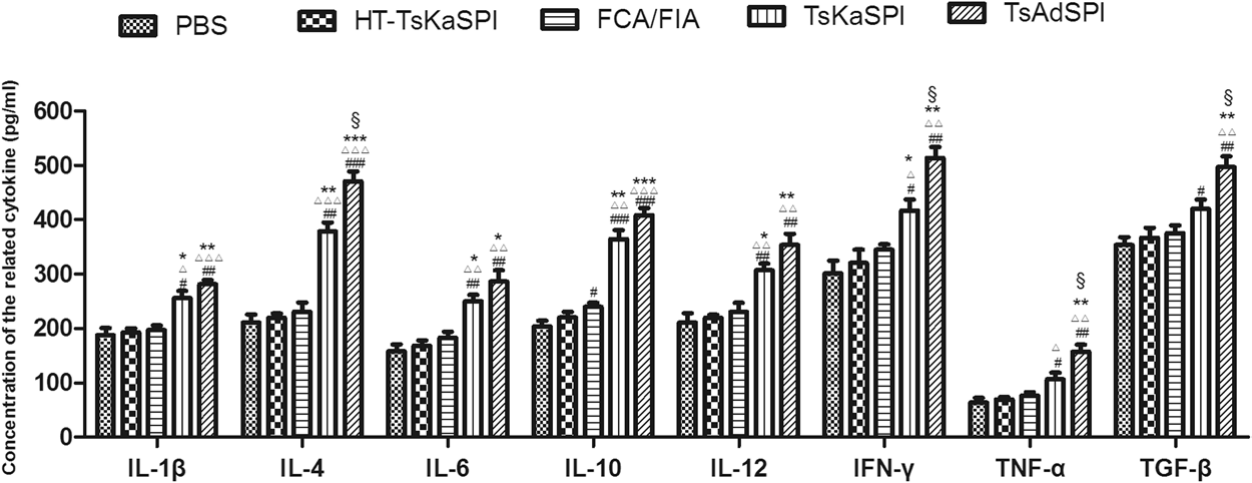
The expression changes of IL-1β, IL-4, IL-6, IL-10, IL-12, IFN-γ, TNF-α and TGF-β were detected by ELISA. Data are shown as mean ± SD of 3 mice per group. ^#^*P* < 0.05, ^##^*P* < 0.01, ^###^*P* < 0.001 versus PBS group; ^△^*P* < 0.05, ^△△^*P* < 0.01, ^△△△^*P* < 0.001 versus HT-TsKaSPI group; ^*^P < 0.05, ^**^P < 0.01, ^***^P < 0.001 versus FCA/FIA group; ^§^*P* < 0.05, ^§§^*P* < 0.01, ^§§§^*P* < 0.001 for TsKaSPI vs TsAdSPI.

The serum of each group was collected and the expression of IL-1β, IL-4, IL-6, IL-10, IL-12, IFN-γ, TNF-α and TGF-β were detected by ELISA. The expression levels of the above cytokines in the TsKaSPI group and TsAdSPI group were significantly higher than those in the PBS group, HT-TsKaSPI group and FCA/FIA group, however, the changes of IL-4, IL-6, IL-10 and IL-12 were significantly higher than IL-1β, IL-4, IL-6, IL-10, IL-12, IFN-γ, TNF-α and TGF-β. And the expression levels of IL-4, IFN-γ, TNF-α, and TGF-β in the TsAdSPI group were significantly higher than those in the TsKaSPI group. But there was no significant difference in the expression of IL-1β, IL-6, IL-10 and IL-12 between the TsKaSPI group and TsAdSPI group (*P* > 0.05) (Fig. 5).

### Adult reduction rate

**Figure 6:**
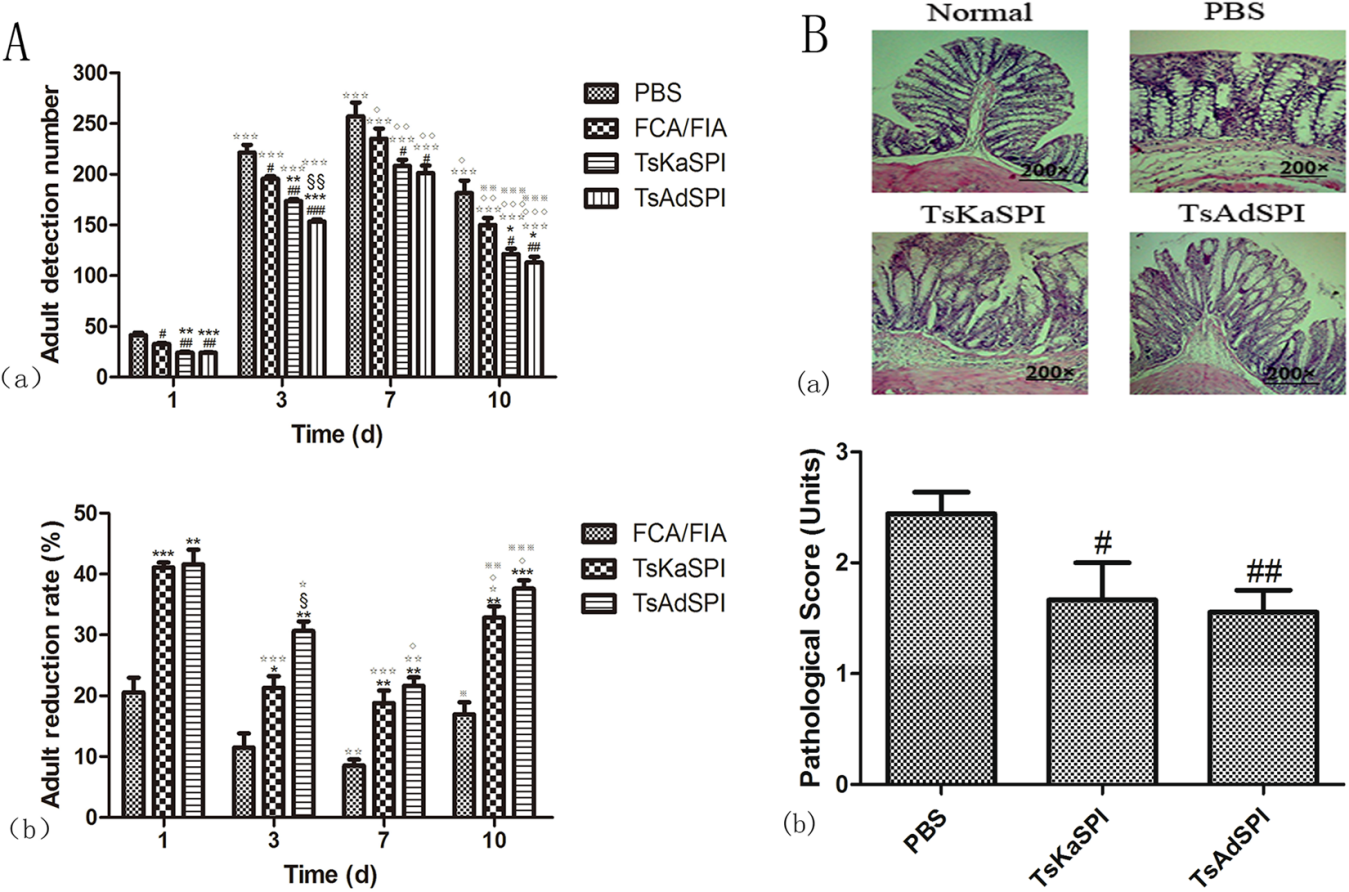
The number of adults detected and the adult reduction rate were showed in (**A**). Data are shown as mean ± SD of 3 mice per group. ^#^*P* < 0.05, ^##^*P* < 0.01, ^###^*P* < 0.001 versus PBS group; ^*^P < 0.05, ^**^P < 0.01, ^***^P < 0.001 versus FCA/FIA group; ^§^*P* < 0.05, ^§§^*P* < 0.0, ^§§§^*P* < 0.001 for TsKaSPI vs TsAdSPI; ^☆^*P* < 0.05, ^☆☆^*P* < 0.01, ^☆☆☆^*P* < 0.001 for each group on the 3rd, 7th, 10th day vs the corresponding group on the 1st day; ^◇^*P* < 0.05, ^◇◇^*P* < 0.01, ^◇◇◇^*P* < 0.001 for each group on the 7th, 10th day vs the corresponding group on the 3rd day; ^※^*P* < 0.05, ^※※^*P* < 0.01, ^※※※^*P* < 0.001 for each group on the 10th day vs the corresponding group on the 7th day. Light micrograph of HE-stained colonic section were showed in (**B**) (a). Scale bar represents 200 μm. And the pathological score were showed in (**B**) (b). TsKaSPI group and TsAdSPI group showed significant improvement than the PBS group.

Mice in each group were infected with 500 *T. spiralis* orally 7 days after the third immunization, and adults were counted on the 1st, 3rd, 7th, and 10th day after infection, and the adult reduction rate was calculated. On the 1st, 3rd, 7th, and 10th day after infection, the number of adults detected in the TsKaSPI group and TsAdSPI group was significantly higher than that in the PBS group and FCA/FIA group, and there was a significant difference between the TsKaSPI group and TsAdSPI group only on the 3rd day (*P* < 0.01), and there was also a significant difference in the number of adults detected in the same group on different days (Fig. 6A(a)).

The adult reduction rate was calculated according to the formula. The results showed that the adult reduction rate of the TsKaSPI group and the TsAdSPI group was significantly higher than that of the FCA/FIA group on the 1st, 3rd, 7th, and 10th day, and there was no significant difference between the TsKaSPI group and TsAdSPI group. At the same time, the adult reduction rate of the same group on different days was also different (Fig. 6A(b)).

### Effect of recombinant protein on intestinal pathological changes

After 5 days of infection, the mice were sacrificed and the intestine was collected for pathological sections, H.E staining was performed. The intestinal pathological changes of mice in each group were observed under the microscope. As shown in the Fig. 6B(a), intestinal wall edema, intestinal epithelial villus rupture, more bleeding spots, inflammatory cell infiltration in the mucosa and submucosa, and uneven arrangement of glands were observed in the PBS group. And the TsKaSPI group and TsAdSPI group showed significant improvement, the mild intestinal wall edema, less bleeding points and cell infiltration were observed. The pathological score which showed in Fig. 6B(b) were used to explain the pathological changes in term of quantitation. From the figure, we observed that in the PBS group (2.44 ± 0.20) was significantly higher than the TsKaSPI group (1.67 ± 0.34) (*P* < 0.05) and TsAdSPI group (1.56 ± 0.20) (*P* < 0.01).

### The expression level of cytokines in cell supernatant

**Figure 7:**
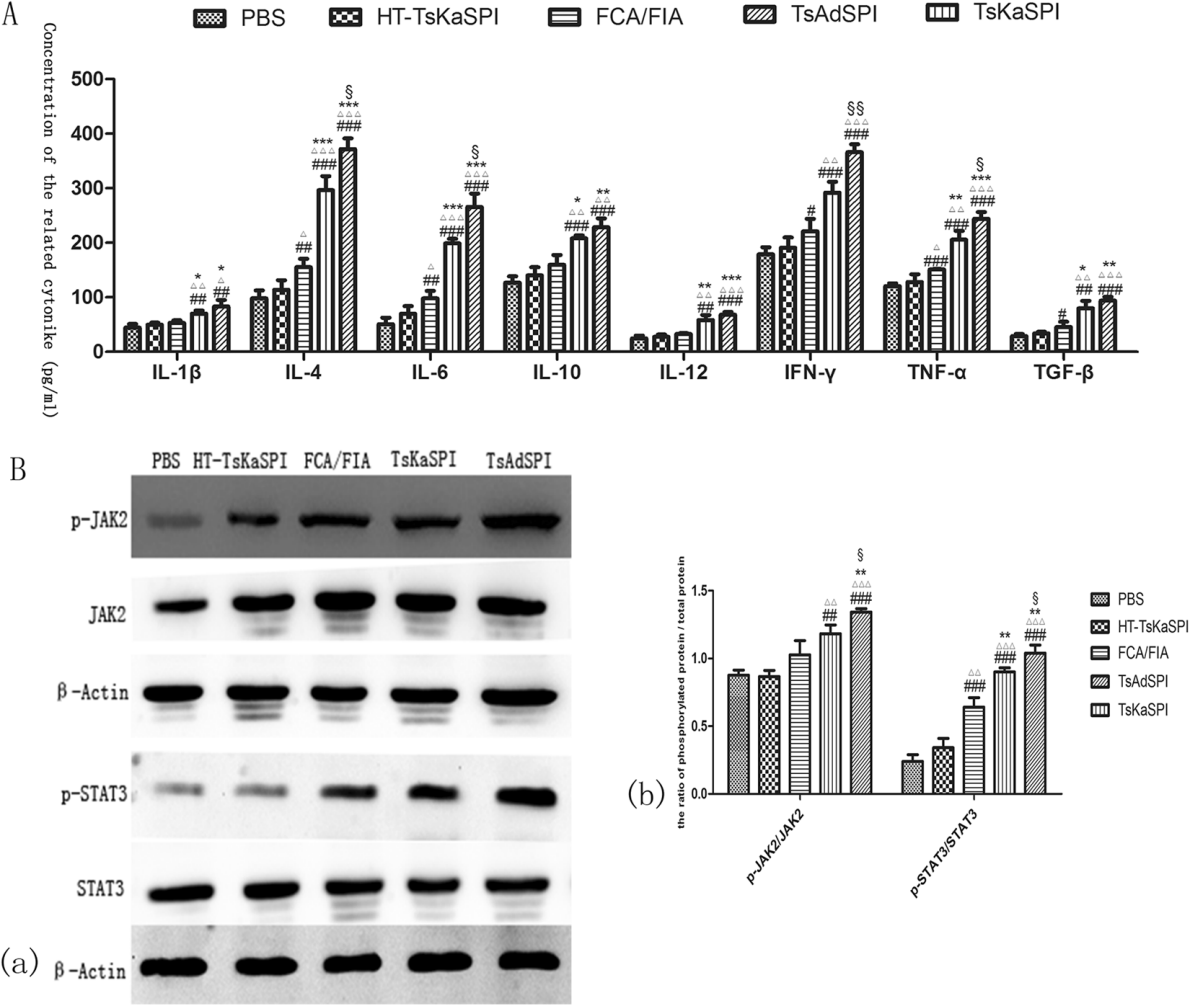
The expression changes of IL-1β, IL-4, IL-6, IL-10, IL-12, IFN-γ, TNF-α and TGF-β in the cell supernatant were showed in (**A**), and the representative gel of the Western Blot were shown in (**B**) (a), and the graph of the quantified band density were also shown in (**B**) (b). Data are shown as mean ± SD of 3 mice per group. ^#^*P* < 0.05, ^##^*P* < 0.01, ^###^*P* < 0.001 versus PBS group; ^△^*P* < 0.05, ^△△^*P* < 0.01, ^△△△^*P* < 0.001 versus HT-TsKaSPI group; ^*^P < 0.05, ^**^P < 0.01, ^***^P < 0.001 versus FCA/FIA group; ^§^*P* < 0.05, ^§§^*P* < 0.01, ^§§§^*P* < 0.001 for TsKaSPI vs TsAdSPI.

ELISA was used to detect the expression of IL-1β, IL-4, IL-6, IL-10, IL-12, IFN-γ, TNF-α and TGF-β in mouse peritoneal macrophages. The expression levels of the above cytokines in the TsKaSPI group and TsAdSPI group were significantly higher than those in the PBS group, HT-TsKaSPI group and FCA/FIA group. Meanwhile compared the TsKaSPI group with the TsAdSPI group, only the concentration of IL-6, IFN-γ, TNF-α were significantly different (*P* < 0.05) (Fig. 7A).

### The effects of two recombinant proteins on JAK2/STAT3 signaling pathway

Total protein from the five groups were extracted and used Western blot to analyze the phosphorylation of JAK2/STAT3. Compared the TsKaSPI (1.18 ± 0.07) and TsAdSPI group (1.34 ± 0.03) with the PBS (0.88 ± 0.04) and HT-TsKaSPI group (0.87 ± 0.05), the results showed that the ratio of p-JAK2 to JAK2 were significantly higher in the front two groups, and the TsAdSPI group was significantly different from the FCA/FIA group (1.03 ± 0.11) (*P* < 0.01). Meanwhile the phosphorylation of STAT3 were significantly higher in the TsKaSPI (0.90 ± 0.03) and TsAdSPI group (1.04 ± 0.06) than that in the PBS (0.24 ± 0.05) (*P* < 0.001), HT-TsKaSPI (0.34 ± 0.07) (*P* < 0.001) and FCA/FIA group (0.64 ± 0.07) (*P* < 0.01). At the same time, the TsKaSPI group was significantly different from the TsAdSPI group (*P* < 0.05). The results showed that both TsKaSPI and TsAdSPI could induce the phosphorylation of JAK2/STAT3 (Fig. 7B) and the complete protein band of p-JAK2, JAK2, p-STAT3, STAT3 were shown in supplemental file.

## Discussion

Some scholars have confirmed that *T. spiralis* infection will produce an immune response characterized by Th1 and Th2 type^16^. Since IgG1 and IgG2a represent Th2 type and Th1 type immune responses, respectively^17^, therefore, we measured the changes of the expression levels of IgG1 and IgG2a in the serum of mice immunized with TsKaSPI and TsAdSPI. The results showed that intraperitoneal injection TsKaSPI and TsAdSPI could increased the concentration of IgG1 and IgG2a in serum of mice, and IgG1 dominated, so we comfirmed that both TsKaSPI and TsAdSPI could induce a mixed Th1/Th2 immune response^18^ with Th2 dominated, it is consistent with expected. Spleen cells proliferation experiments showed that both TsKaSPI and TsAdSPI could induce a strong cellular immune response in the host, so that the body could quickly induce humoral immune and cellular immune response, accelerating the discharge of worms and killing them.

Cytokine is a kind of regulatory protein that regulate immune response^19^. Bojalil *et al*.^20^ showed that IFN-γ acted as a pro-inflammatory cytokine involved in killing *T. spiralis* newbron larvae; Patel *et al*.^21^ demonstrated that IL-4 played an important role in host resistance to *T.s* infection; Beiting *et al*.^22^ showed that IL-10 could inhibit local inflammation in the early phase of *T. spiralis* infection; And the elevated levels of pro-inflammatory cytokines in serum of rats which infected with *T. spiralis* were confirmed by Farid^23^. In this experiment, we measured the expression levels of IL-1β, IL-4, IL-6, IL-10, IL-12, IFN-γ, TNF-α and TGF-β in the serum of mice immunized with TsKaSPI and TsAdSPI, and it was found that the expression of the above cytokines were increased, but with different degrees. This indicated that cytokines have a vital role in regulating the immune response to *T. spiralis* invasion the hosts, and the mixed internal environment formed by pro-inflammatory and anti-inflammatory factors was conducive to protect host from inflammatory damage and inhibited *T. spiralis* parasitization.

T lymphocytes are a major group of immunocompetent cells, which are divided into CD4+ (TH) and CD8+ (TS/TC) cell subsets according to their functions^24^. The subsets are interrelated, mutually promoting and mutually restricting. Ahn *et al*.^25^ found that the activated CD4+CD25+Foxp3+ Tregs in mice which infected with *T. spiralis* was higher than the control group. Ivanoska *et al*.^26^ found that *T. spiralis* could inhibit the pig’s immune function through CD8+ T cells and the inhibitors they secreted, thus evading the attack of host’s immune system. The results of our experiment showed that immunization with TsKaSPI and TsAdSPI could increased the CD4+ T cells and CD4+CD25+Foxp3+ Tregs significantly. Similarly, the CD8+CD28− T cells was also significantly higher. Therefore, it was speculated that the increased number of CD4+ T cells, expression of Foxp3 and number of CD8+CD28− T cells played a critical role in regulating host immune response. Barriga *et al*.^27^ found that the excretory secretion antigen of *T. spiralis* could cause an increase of IL-2, which leaded to an increase of CD8+ cells, resulting in the ratio of CD 4+/CD8+ decreased and inhibited the immune system of host. Our results showed that the CD4+/CD8+ ratio decreased by TsKaSPI and TsAdSPI, but compared with the PBS group, the difference was not significantly. Therefore, the decreased CD4+/CD8+ ratio had little effect on the immunomodulation during *T. spiralis* infection.

Bregs are a group of important immune cells that exert negative immunoregulatory function *in vivo*. Studies have confirmed that Leishmania major and Schistosoma infection could induce Breg cell production. Breg can exert its immunosuppressive function independent of the release of cytokines^28^. CD19+CD5+CD1d^hi^ Breg is currently the most reported type in mouse. However, there are still few reports on whether *T. spiralis* infection can also cause changes of the number of Bregs. Therefore, this experiment analyzes the change of the number of Bregs in the spleen by FCM. The results showed that both TsKaSPI and TsAdSPI significantly increased the CD19+ B cells and CD19+CD5+CD1d^hi^ Breg cells. It has been proved that Breg has a regulatory function on host immune imbalance, but the specific mechanism remains to be further studied.

*T. spiralis* parasitise the intestine in the early phase of infection. Adults feed on the intestinal villi. The larvae penetrate the intestinal villus, and after undergoing four molts, become adult worms^29^. Finally, intestinal tissue would damage and necrosis, hemorrhage and edema, then form ulcers. Calculated the adult reduction rate and compared intestinal pathological changes, the results indicated that TsKaSPI and TsAdSPI might resisted *T. spiralis* invasion and alleviated the pathological damage by regulating the intestinal mucosal immune system.

It is generally believed that the excretory secretion of parasites directly regulates antigen-presenting cells (APC) including macrophages^30^. This study aimed to analyze the effects of TsKaSPI and TsAdSPI on the activity of macrophages *in vivo*. Our study found that both TsKaSPI and TsAdSPI could significantly increase the expression and secretion of IL-1β, IL-4, IL-6, IL-10, IL-12, IFN-γ, TNF-α and TGF-β of mouse macrophages. It indicated that TsKaSPI and TsAdSPI possibly affect the balance between host inflammatory factors by regulating macrophages express and secret cytokines, so as to regulate the host immune response and create an environment conducive to the survival and development of *T. spiralis* in host.

JAK2/STAT3 signaling pathway plays a key role in immune regulation^31^. Some researchers used gene knockout technology to knock out the STAT3 gene in mice, and found that the pro-inflammatory cytokines secreted by macrophages in mice were significantly increased, suggesting that this signaling pathway has a critical role in inducing the transformation of macrophages to alternative activated phenotype^32^. In the process of parasite infection, the JAK2/STAT3 signaling pathway also has a vital role. *Trichinella pseudospiralis* serine protease inhibitors can activate phosphorylation of JAK2/STAT3 in host, and induce macrophage to an alternative activation phenotype to regulates the dynamic balance between pro-inflammatory and anti-inflammatory cytokines^33^. In this study, Western blot was used to detected the effects of TsKaSPI and TsAdSPI on JAK2/STAT3 signaling pathway. The results showed that both TsKaSPI and TsAdSPI could induce the phosphorylation of JAK2/STAT3, thereby transmitting intracellular and extracellular signals and regulating the function of immune cells. We preliminarily speculated that JAK2/STAT3 is one of the signaling pathways in the regulation of host immune responses by *T. spiralis*, which is correlated with the immune escape of *T. spiralis* to some extent.

In order to rule out the effect of LPS components in the recombinant protein on the experimental results, we boiled the recombinant protein for ten minutes, in which the protein component was inactivated, but LPS was still actived. The experimental results showed that the effect of the HT-TsKaSPI group on the experimental results was slightly. This experiment analyzed the effects of TsKaSPI and TsAdSPI on the immune system from various aspects, provided clues for elucidating the immune escape mechanism of *T. spiralis*, and also provided a certain experimental basis for the prevention and treatment of Trichinosis. Therefore, it has certain scientific value. However, for the complex and diverse immuneregulation mechanism remains to be further studied.

## Methods

### Animal and Recombinant protein

**Figure 8:**
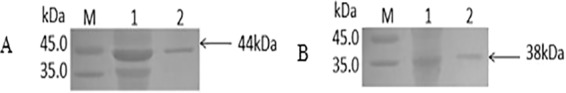
SDS-PAGE analysis of the expression products of before and after purification. M: Protein Marker; 1: Unpurified Protein; 2: Purified Protein.

Male BALB/c mice (SPF) aged 6–8 weeks were purchased from the Animal Center of Harbin Medical University. The study protocol was approved by Northeast Agriculture University Veterinary Research Ethics Committee. And all procedures were strictly in accordance with the guidelines of the Chinese National Institute of Health Guide for the Care and Use of Laboratory Animals. During the whole experiment, the mice were guaranteed to eat and drink freely, and the bedding materials were changed regularly to ensure the excellent feeding environment. And mice were sacrificed using cervical dislocation to alleviate their pain. *T. spiralis* Kazal-type serine protease inhibitors (TsKaSPI) and *T. spiralis* adult serine protease inhibitors (TsAdSPI) recombinant protein were preparation followed the previous method^34^ and stored at −80 °C (Fig. 8).

### Experimental grouping

The laboratory carried out the immune dose optimization experiment, mice were immunized with 20 ug, 50 ug, 100 ug recombinant protein respectively by intraperitoneal injection, the results showed that immunized with 50 ug or 100 ug recombinant protein could promote the concentration of specific IgG in the serum of mice, and there was no significant difference between the 50 ug and 100 ug, so this experiment used 50 ug recombinant protein to immunize mice. We also selected the most commonly used Freund’s adjuvant.

In this experiment, 15 mice were divided into 5 groups: (1) PBS group, immunized mice with 200 ul PBS; (2) HT-TsKaSPI group, the TsKaSPI recombinant protein was diluted to 0.5 ug/ul, then boiled 10 minutes, immunized mice with 100 ul HT-TsKaSPI mixed with 100 ul Freund’s adjuvant; (3) FCA/FIA group, immunized mice with 100 ul PBS and 100 ul Freund’s adjuvant; (4) TsKaSPI group, the TsKaSPI recombinant protein was diluted to 0.5 ug/ul, and immunized mice with 100 ul TsKaSPI mixed with 100 ul Freund’s adjuvant. (5) TsAdSPI group, the TsAdSPI recombinant protein was diluted to 0.5 ug/ul, and immunized mice with 100 ul TsAdSPI mixed with 100 ul Freund’s adjuvant. The mice were immunized three times, each interval of 2 weeks, the first immunization used Freund’s complete adjuvant, the second and third used Freund’s incomplete adjuvant, 7 days after the last immunization, the mice were sacrificed for testing.

### Detection of spleen cells proliferation by CCK-8 method

Prepared the spleen lymphocyte single-cell suspension and inoculated the cell suspension in 96-well plate (100 ul/well), then the plate was pre-incubated in an incubator for 24 hours (37 °C, 5% CO_2_). 10 ul 0.5 ug/ul recombinant protein, ConA and RPMI 1640 were added separately, and stimulated for 48 h in the incubator. Then added 10 ul CCK-8 reaction solution (Beyotime Biotechnology) into each well, and measured the absorbance at 450 nm after incubation for 4 hours in the incubator.

### Flow cytometry detection of CD4+CD25+Foxp3+ Treg cells, CD8+CD28− T cells and CD19+CD5+CD1dhi Breg cells

Prepared the spleen lymphocyte single-cell suspension, each sample was divided into three tubes with 1 × 10^6^ cells per tube. One tube were resuspended in PBS and incubated for 30 min in the dark at 4 °C with FITC anti-mouse CD4 and APC anti-mouse CD25 (Sungene Biotech). Added fixation/permeabilization solution (Invitrogen, USA) to resuspend cells, then incubated for 30–60 min, and washed again. PE anti-mouse Foxp3 (Sungene Biotech) was added after resuspension and incubated for 20 min, washed, then resuspended again for FCM^34^; One tube was resuspended in PBS, added with PE anti-mouse CD8, APC anti-mouse CD28, and incubated for 30 min. Then washed and resuspended for FCM; Other tube was resuspended in PBS, added with FITC anti-mouse CD19, APC anti-mouse CD5 and PE anti-mouse CD1d and then incubated for 30 min. Then washed and resuspended for FCM.

### Detection of IgG subtypes by indirect ELISA

The concentration of recombinant protein was diluted to 10 ug/ml with coating solution, added to the ELISA plate at 100 ul/well, overnight at 4 °C. Washed, then added 100 ul diluted serum sample to the above reaction well, incubated at 37 °C for 1 hour, then washed. Added 100 ul diluted IgG 1 and IgG 2a, incubated for 1 hour. After washing, TMB chromogen solution (Alphabiotech) was added, and added stop solution after 10 min, then measured the absorbance at 450 nm.

### The expression of Th1 and Th2 cytokines detected by ELISA

The serum of mice in each group was collected and cytokines were detected according to the ELISA kit (Alphabiotech) manual.

### Effect of recombinant protein on the adult reduction rate

One week after the third immunization, mice in each group were orally infected with 500 *T. spiralis*, and the mice were sacrificed on the 1st, 3rd, 7th, and 10th day after infection, opened the small intestine longitudinally, removed the contents, placed in normal saline. Then incubated for 3–4 hours at 37 °C, counted the adults under a microscope, and calculated the adult reduction rate according to the formula. The formula is as follows: Adult worm reduction rate (%) = (1 − average adult detection number in the experimental group/average adult detection number in the PBS group) × 100%.

### Effect of recombinant protein on intestinal changes

The intestinal specimens were fixed in 10% paraformaldehyde for several hours and embedded in paraffin sections. Then performed Hematoxylin and eosin (H.E) staining. Histological lesions were assessed using Wallace and Keenan criteria^35^.

### Extraction and purification of peritoneal macrophages

The mice were sacrificed by cervical dislocation, and immersed in 75% alcohol for 3–5 seconds. then placed the mice on dissecting table, and 5 ml pre-cooled PBS was injected into the abdominal cavity along the midline of the abdomen. At the same time, pressed the peritoneal wall for 2–3 minutes. Under sterile conditions, absorbed the liquid in the abdominal cavity to the centrifuge tube. Rinsed the abdominal cavity with the same volume of PBS for 2–4 times until the rinse solution became clear. Centrifuged at 4 °C 250 × g for 10 min, then removed supernatant. The cells concentration adjusted to 5 × 10^6^ cells/ml with RPMI-1640 which containing 10% fetal bovine serum, 100 U/ml penicillin, 10 μg/ml streptomycin. And cultured for 3 h in a 37 °C, 5% CO_2_ incubator. Then washed the cell culture flask 2–3 times with RPMI-1640 medium pre-warmed at 37 °C to removed unattached cells, thus obtaining purified peritoneal macrophage.

### Secretion of cytokines in macrophages detected by ELISA

Cultured the purified peritoneal macrophages for 24 hours. The cell culture supernatant was collected, centrifuged at 2000–3000 r/min for 20 min, and collected the supernatant, then detected the expression of cytokines according to the ELISA kit (Alphabiotech) manual.

### Western blot analysis of the phosphorylation of JAK2/STAT3 pathway in macrophages

Added lysate to fully lyse tissue samples, then centrifuged to obtain the supernatant. Used SDS-PAGE to separated the total protein and blotted the target protein onto a NC membrane. The membrane was blocked for 2 h. Subsequently, incubated with anti-p-JAK2, anti-JAK2, anti-p-STAT3, anti-STAT3 and anti-β-actin (1:4,000 dilution, Bioss) at 4 °C overnight. After washing, incubated with a peroxidase-conjugated secondary antibody (1:5,000 dilution, Bioss) for 1 h. Washed again, then dropped ultrasensitive ECL chemiluminescence reagent (Sangon Biotech) and exposed. Image J software were used to analyze the bands.

### Statistical analysis

All results were expressed as the mean ± standard error. All statistical analysis were performed using GraphPad Prism software and SPSS 13.0 software. *P* < 0.05 was considered statistically significant.

## Supplementary information

Dataset 1
