# Supplementary material for: Regulatory effect of two Trichinella spiralis serine protease inhibitors on the host’s immune system
Source: Sci Rep. 2019 Nov 19;9:17045. doi: 10.1038/s41598-019-52624-5 (PMC6863830; doi:10.1038/s41598-019-52624-5)

Regulatory effect of two *Trichinella spiralis* serine protease inhibitors on the host's immune system

Jingyun Xu [a], Pengcheng Yu [b], Lijia Wu [c], Mingxu Liu [d], Yixin Lu [*]

Address:

[a,b,c,d,*] Heilongjiang Key Laboratory for Zoonosis, *College of Veterinary Medicine, Northeast Agricultural University, 600 Changjiang Street, Harbin 150030, China,* [a] 941606672@qq.com, [b] 827076925@qq.com, [c] 1192256206@qq.com, [d] 1292849000@qq.com.

* Corresponding author. Tel.: +86 451 5519 0729 (Yixin Lu).

* *E-mail address*: luyixin@neau.edu.cn (Yixin Lu).

The full length western blots for the JAK/STAT proteins

p-JAK2

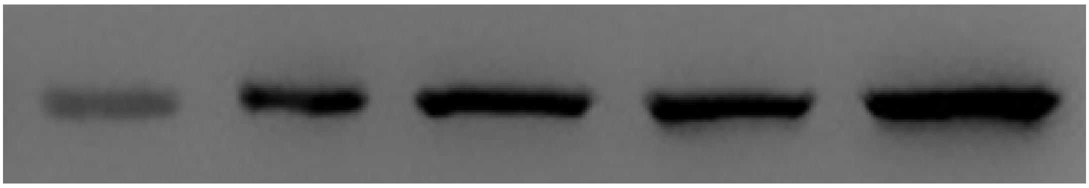

JAK2

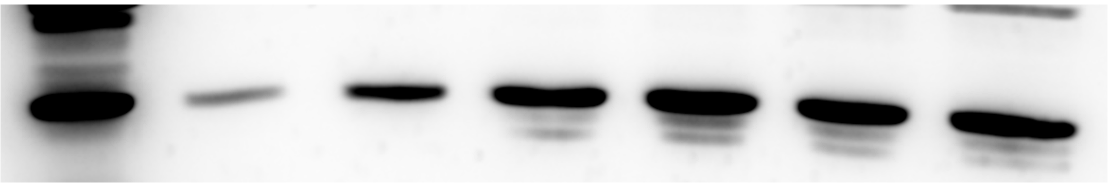

β-Actin

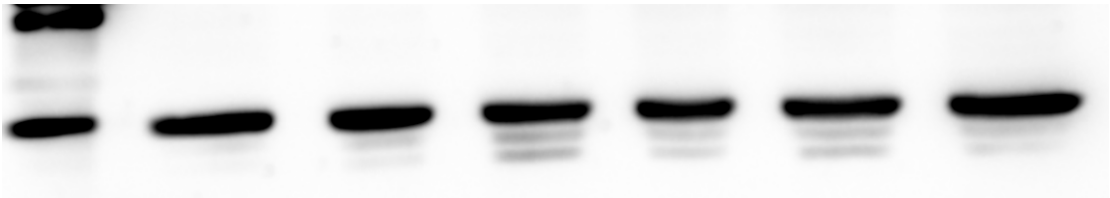

p-STAT3

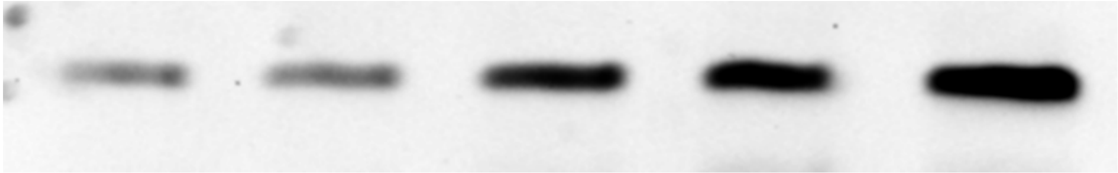

STAT3

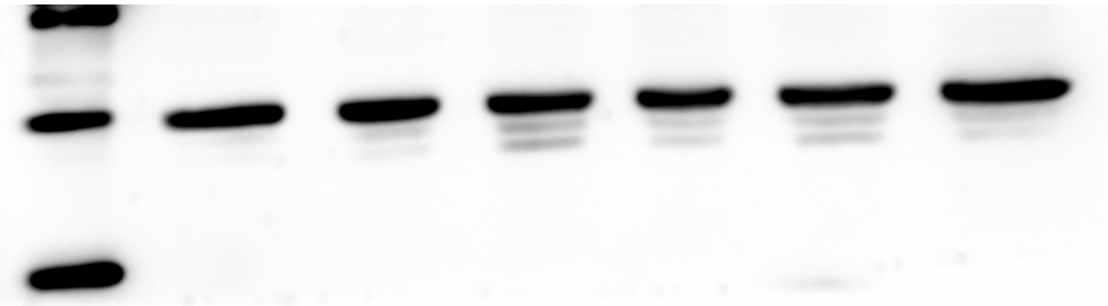

β-Actin

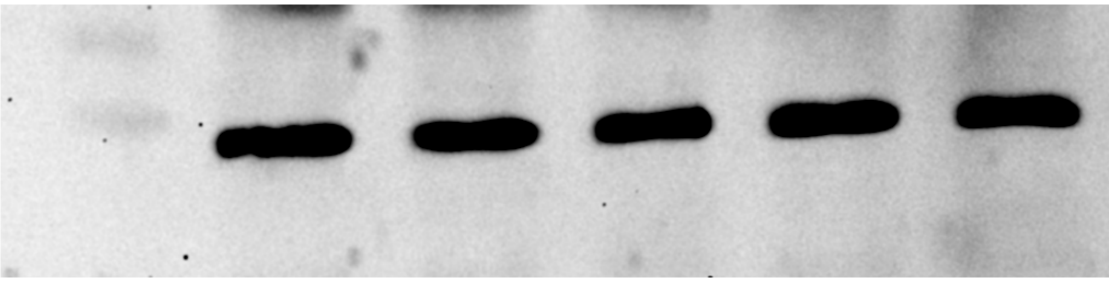

Supplement: Supplementary file 1 — Dataset 1 [file 41598_2019_52624_MOESM1_ESM.pdf]
